# Impact of Repeated Infantile Exposure to Surgery and Anesthesia on Gut Microbiota and Anxiety Behaviors at Age 6–9

**DOI:** 10.3390/jpm13050823

**Published:** 2023-05-12

**Authors:** Xiaoyu Yang, Yan Wu, Xuanxian Xu, Wenzong Gao, Juntao Xie, Zuoqing Li, Xue Zhou, Xia Feng

**Affiliations:** 1 Department of Anesthesiology, The First Affiliated Hospital of Sun Yat-Sen University, Guangzhou 510080, China; 2 Department of Pediatric Surgery, The First Affiliated Hospital of Sun Yat-Sen University, Guangzhou 510080, China; 3 Department of Anesthesiology, Critical Care and Pain Medicine, University of Texas MD Anderson Cancer Center, 1515 Holcombe Blvd., Houston, TX 77030, USA

**Keywords:** anesthesia, infant, gut microbiota, anxiety, development

## Abstract

(1) Background: Preclinical as well as population studies have connected general anesthesia and surgery with a higher risk of abnormal cognitive development, including emotional development. Gut microbiota dysbiosis in neonatal rodents during the perioperative period has been reported, however, the relevance of this to human children who undergo multiple anesthesia for surgeries is unknown. Given the emerging role of altered gut microbes in propagating anxiety and depression, we sought to study whether repeated infantile exposures to surgery and anesthesia affect gut microbiota and anxiety behaviors later in life. (2) Methods: This is a retrospectively matched cohort study comparing 22 pediatric patients of less than 3 years of age with multiple exposures (≥3) to anesthesia for surgeries and 22 healthy controls with no history of exposure to anesthesia. The parent report version of the Spence Children’s Anxiety Scale (SCAS-P) was applied to evaluate anxiety in children aged between 6 and 9 years old. Additionally, the gut microbiota profiles of the two groups were compared using 16S rRNA gene sequencing. (3) Results: In behavioral tests, the p-SCAS score of obsessive compulsive disorder and social phobia were significantly higher in children with repeated anesthesia exposure relative to the controls. There were no significant differences between the two groups with respect to panic attacks and agoraphobia, separation anxiety disorder, physical injury fears, generalized anxiety disorder, and the total SCAS-P scores. In the control group, 3 children out of 22 were found to have moderately elevated scores, but none of them had abnormally elevated scores. In the multiple-exposure group, 5 children out of 22 obtained moderately elevated scores, while 2 scored as abnormally elevated. However, no statistically significant differences were detected in the number of children with elevated and abnormally elevated scores. The data show that repeated anesthesia and surgical exposures in children led to long-lasting severe gut microbiota dysbiosis. (4) Conclusions: In this preliminary study, our findings demonstrated that early repeated exposures to anesthesia and surgical predisposes children to anxiety as well as long-term gut microbiota dysbiosis. We should confirm these findings in a larger data population size and with detailed analysis. However, the authors cannot confirm an association between the dysbiosis and anxiety.

## 1. Introduction

General anesthesia has been seen as a safe means of enabling infants and young children to undergo invasive diagnostic procedures or surgeries. However, concerns over the long-lasting neurotoxic effects of anesthesia and surgery on a developing brain are increasing.

Substantial preclinical studies have indicated that the developing brain is susceptible to anesthetics and surgeries [1]. Recent studies have emphasized that repeated or prolonged neonatal exposure to anesthesia could lead to neurocognitive abnormalities [2,3,4,5]. Additionally, surgery might weigh over anesthesia in affecting cognitive performance in children [6]. The results of clinical studies are mixed. Some human studies of the long-term neurodevelopmental outcomes following general anesthesia in early childhood found no significant differences [7,8], while others were explicitly concerned with persistent changes in behaviors [9]. A systematic review showed that the present clinical evidence suggests a modestly elevated risk of adverse neurodevelopmental outcomes in children who were exposed to anesthesia or surgery during early life, especially for those with multiple exposures [10]. Therefore, the US Food and Drug Administration issued a warning that prolonged (anesthesia duration > 3 h) or repeated exposure to general anesthesia may affect brain development in children under 3 [11]. However, until now, the neuropathological mechanism underlying general anesthesia-induced neurotoxicity has not yet been fully elucidated.

Gut microbiota play a key role in mental health [12]. There are emerging studies emphasizing the importance of early life gut microbiota in shaping future health outcomes [13,14]. It has been reported that alterations to the microbial community early in life increase adverse life events in adulthood [15,16]. Recently, several animal studies revealed that gut microbiota dysbiosis occurs during the perioperative period [17,18,19]. Our recent study revealed that the gut microbiome of neonatal rats underwent changes as a result of repeated anesthesia and surgery exposures. More importantly, after transferring the gut microbiota to young rats treated with antibiotics, they exhibited similar anxiety-like behaviors [20]. Another study using non-human primates found that repeated exposure to sevoflurane results in an anxious phenotype detected later in life [3].

The gut–brain axis is a two-way communication pathway that allows the microbiome to have an impact on anxiety and depression. The possible mechanisms at play include the synthesis of neurotransmitters and the control of the immune system [21]. All emphasize how crucial it is to take the gut microbiome into account when researching mental health disorders. However, there are limited clinical data investigating whether anesthesia would lead to gut microbiota dysbiosis and an anxiety phenotype in children having undergone repeated anesthesia and surgery early in life. To do that, we undertook this retrospective study to elucidate whether repeated anesthesia and surgery can cause the same phenotype and gut microbiota dysbiosis young children. Children aged 6–9 years old, who underwent repeated anesthesia and surgery before they turned 3, were enrolled. The parent report version of Spence Children’s Anxiety Scale (SCAS-P), a suitable assessment for anxiety symptoms, was applied. A Spence Children’s Anxiety Scale (SCAS) is a popular self-report tool for assessing anxiety symptoms in children and teenagers [22]. The SCAS-P is similar to the self-report version in that it asks the same questions, but it is completed by a parent or other adult that is familiar with the child’s behavior [23]. The items evaluate symptoms on six different subscales: physical injury fears, social phobia, panic-agoraphobia, generalized anxiety disorder, and separation anxiety. The SCAS-P is frequently used in both clinical and research settings to evaluate the anxiety symptoms in children and adolescents because it has been shown to have good psychometric properties, including high internal consistency and test–retest reliability [24,25].

In the present study, we aimed to elucidate the possible correlations between early life multiple anesthesia and surgery exposures and changes in gut microbiota and anxiety in young children. By investigating these relationships in young children, a population that may be particularly vulnerable to the long-term effects of anesthesia and surgery, our study provides important insights into the potential risks associated with these common medical procedures.

## 2. Methods

### 2.1. Ethics Approval

The human clinical data were registered in the Chinese Clinical Trial Register (ID: ChiCTR1900022823). Patients’ designated surrogates provided written informed consent according to the Declaration of Helsinki.

Trial registration: Clinical Trial Register ID: ChiCTR1900022823 on the website: http://www.chictr.org.cn/showproj.aspx?proj=38475 (accessed on 26 April 2019). Date of ethics approval: 19 April 2019, registration date: 26 April 2019, start date/date of first subject enrolled: 1 May 2019, and completion date: 1 September 2019.

### 2.2. Human Study

Forty-two children, born between 1 January 2010 and 31 December 2013 in Guangdong, China, were recruited as the cases and controls for this matched case–control study. All participants were residents of the same province. Those exposed to multiple anesthesia (*n* = 21) before the age of 3 were matched to an unexposed control (*n* = 21). The study inclusion criteria were (1) Multiple exposures: children who having undergone more than 3 general anesthesia before the age of 3 for elective surgery; American Society of Anesthesiologists Physical Status 1–2; no medical history of intestinal diseases; 36 weeks’ gestational age or older at birth; (2) Control: age- and sex-matched children with no anesthesia exposure or any medical history of intestinal diseases; 36 weeks’ gestational age or older at birth. All the children and parents were recruited to the hospital. Written consent from parents were obtained. Information related to the procedures performed, the anesthetics used on children and the numbers during the hospital stays in the multiple-exposure group is shown in Supplementary Table S1. Demographics including age, sex, and ASA were collected. Additionally, data were collected on neonatal feeding and nutrition practices, which may have implications for the development of the gut microbiome under study.

### 2.3. Human Behavior Test

The parent report version of Spence Children’s Anxiety Scale (SCAS-P) in Chinese was applied to evaluate anxiety in children aged 6–9 years. The SCAS-P has been proven reliable and valid for use with mainland Chinese children [26]. The SCAS-P consists of 38 items to evaluate specific symptoms of anxiety disorders, including obsessive compulsive disorder (6 items), social phobia (6 items), panic attack and agoraphobia (9 items), separation anxiety disorder (6 items), physical injury fears (5 items), and generalized anxiety disorder (6 items). Each item is rated on a 4-point scale (from “0 = never” to “3 = always”). The total SCAS score is the sum of all the sub-item scores. For girls, an elevated score is defined as total SCAS score above 28–29, while the score above 38 is thought of as abnormally elevated. For boys, an elevated score is defined as the total SCAS score above 26, while a score above 35–36 is thought to be abnormally elevated. On a 4-point scale, parents rated their children’s anxiety symptoms, with a higher score indicating more anxiety. The questionnaires were completed at the hospital, and parents were asked to be honest and not share their responses with others. To ensure confidentiality, a numeric code was used to replace participants’ personal information.

### 2.4. Sample Collection and Storage

The participants lay on their side with their knees drawn up towards their chest to collect a rectal sample. A Dacron swab (Thermo Fisher Scientific, Waltham, MA, USA) was inserted into the rectum 1–2 cm past the anal verge and gently rotated for several seconds to collect a sample of fecal material. The swabs were then placed in a sterile empty tube, put on ice, and immediately transferred to −80 °C for further testing [27].

### 2.5. DNA Extraction and Quantification of Fecal Bacteria

Human microbial DNA from the rectal swab was extracted using the DNA extraction kit using FastDNA^TM^ SPIN KIT FOR SOIL (Qbiogene, Carlsbad, CA, USA). The concentration and purity were measured by the NanoDrop One (Thermo Fisher Scientific).

### 2.6. Sequencing Data Processing

Sequencing libraries were generated using the NEBNext^®^ UltraTM II DNA Library Prep Kit for Illumina^®^ (New England Biolabs, Ipswich, MA, USA) following the manufacturer’s recommendations. Library quality was assessed using a Qubit@ 2.0 Fluorometer (Thermo Fisher Scientific, Ipswich, MA, USA). The library was then sequenced on an Illumina Nova6000 platform (Illumina, San Diego, CA, USA), generating 250 bp paired-end reads. Raw data quality was controlled using Fastp (an ultra-fast all-in-one FASTQ preprocessor, version 0.14.1) with a sliding window (-W 4 -M 20). Primers were removed using the cutadapt software according to primer information at the beginning and end of the sequence to obtain paired-end clean reads. Paired-end clean reads were merged using the usearch-fastq_mergepairs (V10) according to the overlap relationship between the paired-end reads; when an overlap of at least 16 bp was found between the reads generated from the opposite ends of the same DNA fragment and the maximum mismatch allowed in overlap region was 5 bp, spliced sequences were called raw tags. Fastp (version 0.14.1) was used again to control the raw data quality by the sliding window (-W 4 -M 20) to obtain paired-end clean tags.

### 2.7. V4-5 16S rRNA Gene Region Sequencing and Analysis

For sequencing, the 16S rRNA gene was PCR amplified using primers U515F 5′-GTGCCAGCMGCCGCGGTAA-3′ and U907R 5′-CCGTCAATTCMTTTRAGTTT-3′ targeting the V4–V5 hypervariable regions with 12bp barcode. The V4–V5 region has the advantage of allowing the detection of both bacterial and archaeal taxa, which can provide a more comprehensive understanding of the microbial community. Furthermore, the V4–V5 region has been shown to have low levels of intragenomic variation, which decrease the possibilities of sequencing errors or chimeric sequences. Sequences with ≥97% similarity were assigned to the same OTU. Sequence analyses were performed by usearch software (V10). Alpha diversity is applied in analyzing the complexity of species diversity, while beta diversity analysis was used to evaluate the differences of samples in species complexity using analysis of similarity (ANOSIM). The weighted UniFrac distance matrices visualized by principal coordinate analysis (PCoA) were calculated. LDA Effect Size (LEfSe) analysis, which emphasizes both statistical significance and biological relevance, was used to find the biomarker of each group by R software (Version 3.4.4, R Foundation for Statistical Computing, Vienna, Austria).

### 2.8. Statistics

GraphPad Prism (Version 8.0, GraphPad Software, Inc., San Diego, CA, USA) was used for all statistical analysis and the creation of figures. The test of normality was performed using the Shapiro–Wilk test. Normally distributed values are presented as mean ± SEM, while non-normally distributed variables are shown as the median and interquartile range. Comparisons of the means of two independent groups were performed using the Student *t*-test or Mann–Whitney U test, while comparisons of the two paired groups were using the paired *t*-test or Wilcoxon matched-pairs signed rank test, as appropriate. Chi-square analyses were conducted on the SCAS-P to examine the differences in the numbers of children at risk for anxiety and at risk for depression.

For microbiota analysis, we calculated the alpha diversity using QIIME. Beta diversity analysis was performed using an analysis of similarity (ANOSIM) [28,29]. Weighted UniFrac distance matrices were calculated and visualized by PCoA [30]. We used R software to generate a heat map based on the relative abundance of species at each classification level. We considered *p*-values of 0.05 or less to be statistically significant.

The sample size was estimated by the outcome of the preliminary experiment using Z test (pooled): the incidence of elevated of social phobia of SCAS-P in the control group was 8.3% (1/12), while the incidence of multiple-exposure group was 50.0% (6/12), with a power of 0.8, type Ⅰ error of 0.05, and a sample size of *n* = 18. We added three extra children per group to accommodate for potential drop outs from the experiment. Thus, *n* = 21 per group was the calculated sample size.

## 3. Results

### 3.1. Repeated Anesthesia/Surgery Exposures Induced Changes in Gut Microbiome of Human Subjects

**Figure 1:**
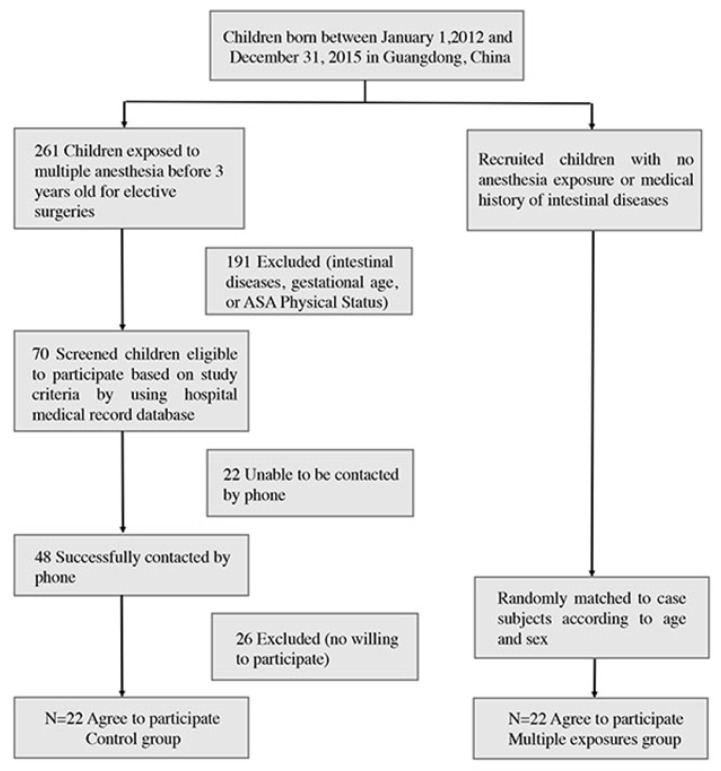
Diagram illustrating the flow of case–control matching process.

The case-match flow chart is shown in Figure 1. The demographics of participant matched pairs exposed and unexposed to anesthesia were listed in Table 1. After conducting a thorough analysis of the demographic data, we found no statistically significant differences in age at the time of testing, the American Society of Anesthesiologists physical status classification (ASA-PS) at first exposure to anesthesia, neonatal feeding practices, or the nutritional status between the two groups. These results suggest that any observed differences in microbiome composition between the groups cannot be attributed to these demographic or medical factors, and support the validity of our comparisons between the groups.

**Figure 2:**
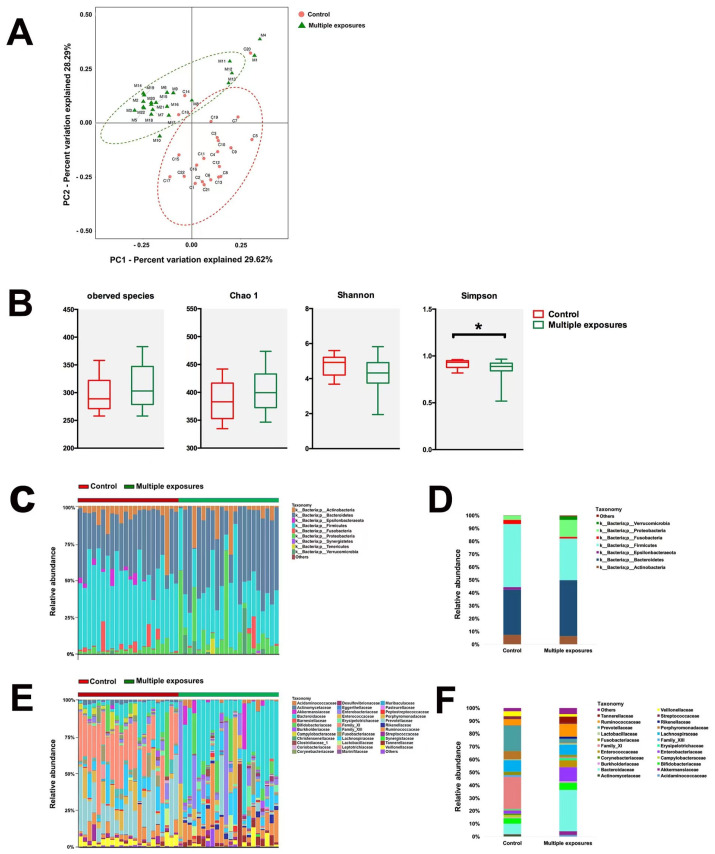
Configuration of the gut microbiome of human fecal pellets. (**A**) PCoA analysis of weighted UniFrac distance representing beta−diversity of the gut microbiota in children from both the control group (Red) and multiple-exposure group (Green). PCoA plots explain 29.62% and 28.29% of the variance of weighted UniFrac distances from individual subjects. (**B**) Indices representing the alpha−diversity of the gut microbiota between the two groups. The analysis of α−diversity indices proved no statistical difference between two groups in observed species, Chao 1, and Shannon (*P*_observed species_ = 0.11, *P*_Chao1_ = 0.09, *P*_Shannon_ = 0.06, *p* > 0.05), while a significant difference was shown in Simpson (*P*_Simpson_ = 0.024). *: *p* < 0.05. Differences can be seen in the microbiota composition at the phylum and family levels in humans (**C**–**F**). Bar graphs show the individual child (**C**) and mean (**D**) relative abundance of the major phyla in rats of both the control group and multiple-exposure group. Bar graphs show an individual child (**E**) and mean (**F**) relative abundance of the major families. Red: control group; Green: multiple-exposure group.

Rectal samples from both groups were collected and analyzed. The principal component of the gut microbiome in both groups was analyzed by PCoA based on the weighted UniFrac distance on operational taxonomic units (OTUs) (Figure 2A). The first two axes are represented with principal coordinate axis 1 (29.6% variability) and principal coordinate axis 2 (28.29% variability). PERMANOVA analyses found sufficient evidence to reject the hypothesis that children with multiple anesthesia exposure were similar to those without exposure (Figure 2A). The present results demonstrated that the overall composition of the gut microbiota community was different between the two groups of children. The analysis of α-diversity indices showed no statistical difference between the groups in observed species (Chao 1, and Shannon (Figure 2B, *P*_observed species_ = 0.11, *P*_Chao1_ = 0.09, *P*_Shannon_ = 0.06, *p* > 0.05). However, a significant difference was observed in Simpson (*P*_Simpson_ = 0.024). The results demonstrated that the OUT richness, evenness, and rare species were slightly different between the two groups.

The abundance of bacteria is represented at the phylum (Figure 2C,D) and family levels (Figure 2E,F). In the phylum-level, the significant changes in relative abundance were found in Firmicutes and Proteobacteria. The relative abundance of Firmicutes significantly decreased (*p* = 0.0003) in control group (0.49 ± 0.03) compared to the multiple-exposure group (0.32 ± 0.03). However, Proteobacteria were significantly higher (*p* = 0.017) in the control group (0.03 ± 0.0006) compared to the multiple-exposures group (0.13 ± 0.04). At the family-level, the combination of bacteria in two groups changed a lot from gross observation. In terms of the top four predominant bacteria, Family_XI (0.25 ± 0.03), Prevotellaceae (0.19 ± 0.03), Lachnospiraceae (0.09 ± 0.02), and Bacteroidaceae (0.08 ± 0.02) were found in the control group. However, Bacteroidaceae (0.32 ± 0.04), Enterobacteriaceae (0.11 ± 0.04), Ruminococcaceae (0.10 ± 0.02), and Lachnospiraceae (0.09 ± 0.02) were topped in the multiple-exposure group.

**Figure 3:**
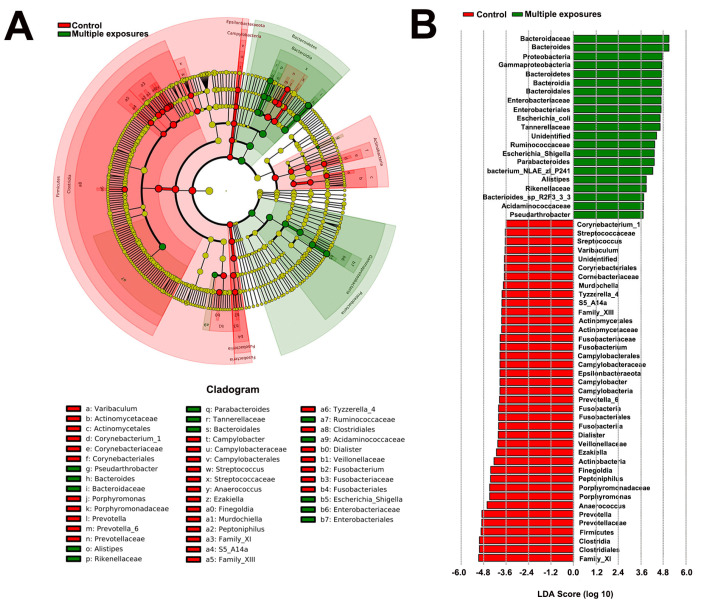
Differentially abundant bacterial taxa between the control group and multiple−exposure group. (**A**) Cladogram generated by LEfSe indicating differentially abundant bacterial taxa in different phylogenetic levels. Regions in red indicate taxa enriched in the control group while regions in green indicate taxa enriched in multiple-exposure group. (**B**) LDA scores shown in the bar graph. Bacterial taxa which were significantly enriched in the multiple−exposure group (LDA score > 3.6) or control group (LDA score < −3.6) were detected by LEfSe analysis (*p* < 0.05).

An LEfSe comparison of gut microbiota between the two groups was shown in Figure 3. The taxonomic cladogram showed the greatest differences in taxa between the two groups (Figure 3A). A number of key phylotypes as microbiological markers were identified at different phylogenetic levels. Clostridia (belonging to phylum Frimicutes) could be used as a microbiological marker to differentiate the control group from the multiple-exposure group. In addition, Bacterioidia (candidate of phylum Bacteridetes) could be used to differentiate the multiple-exposure group from the control group. Additionally, the taxa meeting an LDA score threshold of >3.6 are listed (Figure 3B).

### 3.2. SCAS-P Anxiety Test Revealed Slight Changes in Children with Repeated Anesthesia Exposure

**Figure 4:**
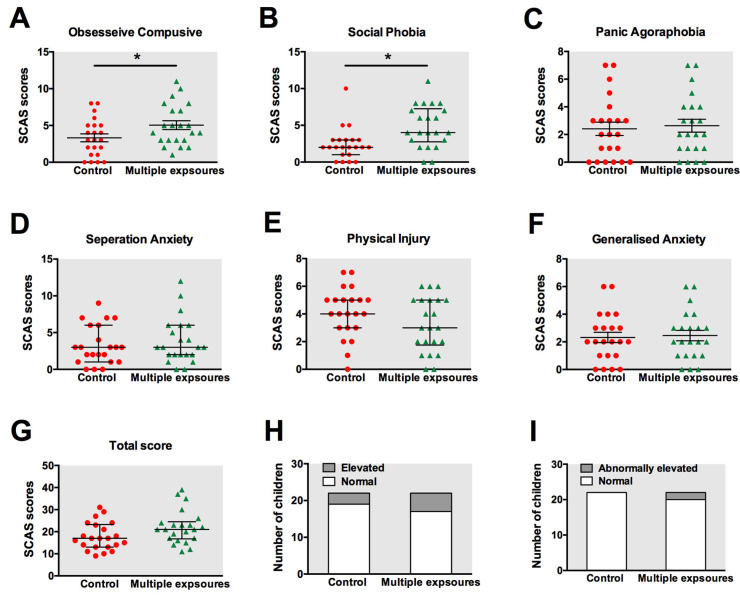
Anxiety status in children tested by SCAS. The scores of obsessive compulsive disorder (**A**), social phobia (**B**), panic attacks, and agoraphobia (**C**), separation anxiety disorder (**D**), physical injury fears (**E**), generalized anxiety disorder (**F**), and the total score (**G**) were calculated and compared between the control group and multiple-exposure group. The means and standard error for each outcome measure are presented. The number of children with elevated (**H**) or abnormally elevated scores (**I**) were also evaluated. *: *p* < 0.05. Red: control group; Green: multiple-exposure group.

The scores of both sub-items and the grand total were calculated and compared between the two groups. The means and standard error of the mean for each outcome measure were presented in Figure 4A–G. The results revealed statistically significant differences in the SCAS-P scores between children with and without anesthesia exposure with respect to obsessive compulsive disorder (Figure 4A, *p* = 0.04) and social phobia (Figure 4B, *p* = 0.01). However, no significant differences were found between the two groups in terms of panic attacks and agoraphobia (Figure 4C, *p* = 0.72), separation anxiety disorder (Figure 4D, *p* = 0.66), physical injury fears (Figure 4E, *p* = 0.21), generalized anxiety disorder (Figure 4F, *p* = 0.83), and the total score (Figure 4G, *p* = 0.09).

We also counted the number of children with elevated (Figure 4H) or abnormally elevated scores (Figure 4I). In the control group, 3 children out of 22 were found to have elevated scores, but none obtained abnormally elevated scores. In the multiple-exposure group, 5 children out of 22 obtained elevated scores, while 2 scored as abnormally elevated. However, no statistically significant differences were detected in the number of children with elevated and abnormally elevated scores using chi-square analysis.

## 4. Discussion

In the present study, we conducted a retrospective matched cohort study to examine how the intestinal microbiome composition is affected by multiple surgical and anesthesia exposures and how these affect younger children’s anxious symptomatology. We found some degree of anxious emotional disorders as well as gut microbiota dysbiosis in 22 children who underwent more than three rounds of anesthesia and surgeries before they turned three. To our knowledge, the microbiota composition of children with repeated anesthesia and surgery exposure had not been reported.

The peak synaptogenesis occurs from birth to 3 years of age in human brains [31]. This period is also most vulnerable to anesthetic induced neurotoxicity [32]. Therefore, we enrolled children who were exposed to multiple anesthesia in their first 3 years of life. It is reported that the first microbial inoculum is formed by the maternal microbiota, and after birth, the diversity of microorganisms rises and converges to an adult-like microbiota by the end of the first 3–5 years of life [33,34]. At the time of the study, the children were aged 6–9 years old, which means that the gut microbial diversity has become stable. Gut microbiota are not only intrinsically different, but are also affected by environmental factors, including housing conditions, dietary impacts, genetic background, and founder effects [35]. Furthermore, the human gut is a relatively complicated ecosystem that could be influenced by several factors such as age, genetics, diet, and geographic variations [36]. Therefore, we performed an age- and sex-matched case–control study and enrolled children who were raised in the same region to retain consistent demographic and clinical characteristics. Additionally, in this study, we attempted to rule out any potential influences on the gut microbiota, such as geographic location, breastfeeding practices during infancy, dietary habits, etc. When two groups’ neonatal feeding and nutrition were evaluated, no statistically significant differences were discovered. The main points of distinction between the two groups of children were whether they had been hospitalized multiple times, the number of surgeries and anesthesia they received, as well as the history of hospitalization medication.

It has been reported for current preclinical studies and a portion of clinical trials that exposure to general anesthesia and surgery during early brain development periods may be linked to long-term alterations in cognition and behavior [37]. A clinical study carried out at the Mayo Clinic revealed that children with repeated exposure to general anesthesia before the age of 2 have increased risk for the later development of attention-deficit/hyperactivity disorder (ADHD) [38]. Another observational cohort study claimed that children who had general anesthesia and surgery under the age of 5 have significantly higher risk of mental disorders [39]. Our recent research indicated that early repeated exposure to anesthesia and surgery causes anxiety emotion behaviors in rats [20]. Another study showed that the inhalation of anesthetic can cause social behavioral alterations and an anxious phenotype in young non-human primates [40]. That is why we focused on the anxiety phenotype. At this stage, there are reliable tools for behavior tests that can be used, such as the SCAS_P. SCAS-P has been previously proved to test the suitable for assessing anxiety symptoms in children and adolescents from mainland China [26]. SCAS can detect specific anxiety disorders, and has been found to have strong internal consistency, influenced by different moderators [41]. It has good psychometric properties which lead to the correct and early diagnosis of anxiety disorders. It was previously reported that SCAS-P is an adequate measurement to assess anxious symptomatology in young children [42]. According to our results, we found statistical differences between the two groups in terms of social phobia and obsessive compulsive behavior. The SCAS’s social phobia subscale contains questions that measure the child’s anxiety about and avoidance of social situations as well as their anxieties about looking ridiculous or weak in front of others [43]. Items on the obsessive compulsive behavior subscale measure the child’s propensity for engaging in repetitive, compulsive actions or thoughts [43]. The SCAS’s subscales for obsessive compulsive behavior and social phobia can both reveal important details about the child’s anxiety symptoms and may be helpful in identifying kids who might benefit from additional assessment or treatment for anxiety disorders. However, no significantly difference was found between the groups with respect to the number of children who obtained elevated or abnormally elevated scores, which might be caused by the limited number of participants.

Previous research has suggested that intestinal dysbiosis arising from diverse determinants in the formative years of life may escalate the vulnerability to nervous system development and psychiatric ailments, while engendering persistent repercussions. Autism spectrum disorders (ASDs) and attention-deficit/hyperactivity disorder (ADHD) frequently emerge at this stage [44]. Here, we figured out that early life repeated anesthesia exposure can cause gut microbiota dysbiosis at the age of 6–9. According to our data, the potential mechanisms for the difference in the intestinal microbial flora of the two patient groups are listed. Firstly, children who have many surgical and anesthesia exposures may experience a lot of stress. Stress has been shown to alter the composition of gut bacteria via stress hormones, inflammation, and autonomic alterations [45]. Meanwhile, many drugs, including anesthetics, were administered during the hospitalization. The composition and function of the gut microbiome may change during drug metabolism [46].

Clinically, the microbiota composition of children having experienced repeated surgery and anesthesia exposure, to date, has not been reported. However, in preclinical studies, anesthetics exposure has been shown to alter gut microbiota (GM) profiles. A study performed by Wang et al., revealed that juvenile rats showed changes in gut microbiota after the neonatal exposure to single isoflurane [18]. They found that the abundance of Firmicutes and Proteobacteria were significantly increased in exposed rats compared to controls. Our recent research also indicates the potential of anesthetics to induce gut microflora dysbiosis in rats [20]. We discovered the difference to be that, in exposed rats, Proteobacteria increased while the relative abundance of the phyla Firmicutes decreased. Interestingly, consistent with the animal study, we found in the present study that the relative abundance of Proteobacteria was significantly increased in the multiple-exposure group, while the abundance of Firmicutes was significantly decreased. One major depression disorder (MDD) study also found that patients had higher Proteobacteria and lower Firmicutes [47]. It is obvious to note that rat and human gut microbiota differ. Because of the inherent differences between the gut microbiota of rats and humans, environmental factors such as housing conditions, dietary influences, genetic make-up, and founder effects can also have an impact [35]. However, even so, we noticed comparable alterations in both humans and rats. The long-lasting changes of gut microbiota observed in both rats and humans might offer us a new insight into the pathogenesis of multiple-anesthesia- and surgery-induced neurotoxicity in developing brains. To the best of our knowledge, this is the first report on the composition of microbiota in children who have been exposed to repeated anesthesia and surgeries. Our results highlight the need for further investigation into the potential impact of these procedures on gut microbiota and the overall health and development of young children. Further studies could help identify potential preventive strategies or treatments to mitigate the adverse effects of repeated exposure to anesthesia and surgery in early childhood.

There are limitations in the current study. Firstly, the number of children enrolled in our study who accepted repeated surgery and anesthesia is limited. The relatively small number of subjects limits the generalizability of the present study’s findings. Future studies with a large sample size are still warranted. Secondly, human data might be significantly less relevant compared to the animal data. Children recruited in the study were raised with more complex factors, such as diet, environmental exposure, maternal carryover, and different disease status. Although we tried to exclude some of these factors, there were still factors that could be contributory to the differences between groups in the gut microbiota and their behaviors other than exposure to anesthetics at an early life stage. Thirdly, our present study could not fully indicate the association between gut microbiome dysbiosis and the functional relevance. The authors were unable to support a link between anxiety and dysbiosis, however. It is necessary to conduct additional research on these modifications to determine the precise connection between the gut microbiome and the phenotype of anxiety.

Our exploratory investigation yielded compelling evidence suggesting that frequent and early exposure to anesthetic and surgical procedures may lead to long-term anxiety and chronic gut microbiota dysbiosis. Our findings underscore the importance of considering the potential adverse effects of anesthesia and surgery, particularly in young children who may be more vulnerable to these effects due to their developing gut microbiota and neurodevelopmental processes. The relationship between gut microbiota composition and exposure to anesthesia and surgery remains poorly understood, however, and future studies are needed to establish the temporal and causal links between these factors. Moreover, further research is necessary to assess the utility of the gut microbiome as a potential biomarker for predicting the risk of anxiety and other adverse outcomes following anesthesia and surgery. Despite these limitations, our study highlights the potential value of investigating the role of the gut microbiome in mediating the long-term effects of early exposure to anesthesia and surgery.

## Figures and Tables

**Table 1:** Demographics of participant-matched pairs exposed and unexposed to anesthesia.

Characteristics	Multiple-Exposure Group	Control Group	*p*
Age at testing, mean (SED)	7.13 (0.18)	7.15 (0.18)	0.97
Exposure No., mean (SED)	3.86 (0.21)	0 (0)	<0.001
Sex, No. (%)			
Male	15	15	>0.99
Female	7	7	>0.99
ASA-PS at first exposure			
I	22	22	>0.99
II	0	0	>0.99

## Data Availability

The 16SrRNA gene sequence data have been submitted to NCBI under Bioproject: PRJNA597811 and can be accessed at https://www.ncbi.nlm.nih.gov/bioproject/597811, accessed on 10 April 2023.

## Supplementary Materials

The following supporting information can be downloaded at: https://www.mdpi.com/article/10.3390/jpm13050823/s1, Table S1: Procedures performed and anesthetics used on children in Multiple exposures group.
