# Supplementary material for: Impact of Repeated Infantile Exposure to Surgery and Anesthesia on Gut Microbiota and Anxiety Behaviors at Age 6–9
Source: J Pers Med. 2023 May 12;13(5):823. doi: 10.3390/jpm13050823 (PMC10221151; doi:10.3390/jpm13050823)
Supplement: Supplementary file 1 [file jpm-13-00823-s001.zip › jpm-2262200-supplementary.pdf]

Participate No.	Date of Birth	Gender	Anesthetics used	Procedures performed before 36 mo		
				Surgery types	Numbers	Duration of hospital stay
M1	2012/04/27	Female	Propofol, Fentanyl, Remifentanil, cis-atracurium	Laryngeal papillomatosis resection procedures	4	
M2	2013/03/28	Male	Propofol, Sevoflurane, Fentanyl, Remifentanil, cis-atracurium	Testis and urinary tract procedures	4	
M3	2012/04/19	Male	Propofol, Sevoflurane, sufentanil, Remifentanil, cis-atracurium	Testis and urinary tract procedures	3	
M4	2012/09/08	Male	Propofol, Sevoflurane, Fentanyl, Remifentanil, cis-atracurium	Kidney and urinary tract procedures	6	
M5	2012/07/25	Male	Propofol, Sevoflurane, Fentanyl, Remifentanil, cis-atracurium	Laryngeal papillomatosis resection procedures	3	
M6	2012/06/05	Male	Propofol, Sevoflurane, Sufentanil, Remifentanil, cis-atracurium	Laryngeal papillomatosis resection procedures and circumcision	4	
M7	2011/01/21	Female	Propofol, Sevoflurane, Fentanyl, Sufentanil, Remifentanil, cis-atracurium	Tonsillectomy and adenoidectomy	3	
M8	2010/10/29	Male	Propofol, Sevoflurane, Fentanyl, Remifentanil, cis-atracurium	Finger and hand surgeries	3	
M9	2012/11/28	Female	Propofol, Sevoflurane, Fentanyl, Sufentanil, Remifentanil, cis-atracurium	Foot and knee surgeries	4	
M10	2011/10/23	Male	Propofol, Sevoflurane, Fentanyl, Remifentanil, cis-atracurium	Foot and knee surgeries	4	
M11	2011/06/24	Male	Propofol, Sevoflurane, Sufentanyl, Remifentanil, cis-atracurium	Laryngeal papillomatosis resection procedures	5	
M12	2012/09/16	Male	Propofol, Sevoflurane, Fentanyl, Remifentanil, cis-atracurium	Plastic surgeries for repairing burn	3	
M13	2011/09/16	Male	Propofol, Sevoflurane, Fentanyl, Remifentanil, cis-atracurium	Foot and knee surgeries	3	

M14	2012/02/22	Male	Propofol, Sevoflurane, Fentanyl, Remifentanil, cis-atracurium	Procedures on mouth/tongue and cleft lip and palate repair	4
M15	2012/06/22	Female	Propofol, Sevoflurane, Fentanyl, Remifentanil, cis-atracurium	Foot and knee surgeries	3
M16	2011/04/27	Male	Propofol, Sevoflurane, Fentanyl, Remifentanil, cis-atracurium	Laryngeal papillomatosis resection procedures	3
M17	2012/07/25	Male	Propofol, Sevoflurane, Fentanyl, Sufentanil, Remifentanil, cis-atracurium	Kidney and urinary tract procedures	6
M18	2010/10/16	Male	Propofol, Sevoflurane, Fentanyl, Remifentanil, cis-atracurium	Urinary tract procedures	3
M19	2012/09/26	Female	Propofol, Sevoflurane, Fentanyl, Remifentanil, cis-atracurium	Upper airway procedures	5
M20	2011/07/20	Male	Propofol, Sevoflurane, Fentanyl, Sufentanil, Remifentanil, cis-atracurium	Kidney and urinary tract procedures	5
M21	2010/01/11	Female	Propofol, Sevoflurane, Fentanyl, Remifentanil, cis-atracurium	Procedures on mouth/tongue and cleft lip and palate repair	3
M22	2012/08/27	Female	Propofol, Sevoflurane, Sufentanyl, Remifentanil, cis-atracurium	Laryngeal papillomatosis resection procedures	4

Supplementary Table S1. Procedures performed and anesthetics used on children in Multiple exposures group.
